# Real time electrocardiogram QRS detection using combined adaptive threshold

**DOI:** 10.1186/1475-925X-3-28

**Published:** 2004-08-27

**Authors:** Ivaylo I Christov

**Affiliations:** 1 Center of Biomedical Engineering, Bulgarian Academy of Sciences, Acad. G. Bonchev str., blok 105, 1113, Sofia, Bulgaria

## Abstract

**Background:**

QRS and ventricular beat detection is a basic procedure for electrocardiogram (ECG) processing and analysis. Large variety of methods have been proposed and used, featuring high percentages of correct detection. Nevertheless, the problem remains open especially with respect to higher detection accuracy in noisy ECGs

**Methods:**

A real-time detection method is proposed, based on comparison between absolute values of summed differentiated electrocardiograms of one of more ECG leads and adaptive threshold. The threshold combines three parameters: an adaptive slew-rate value, a second value which rises when high-frequency noise occurs, and a third one intended to avoid missing of low amplitude beats.

Two algorithms were developed: Algorithm 1 detects at the current beat and Algorithm 2 has an RR interval analysis component in addition.

The algorithms are self-adjusting to the thresholds and weighting constants, regardless of resolution and sampling frequency used. They operate with any number *L *of ECG leads, self-synchronize to QRS or beat slopes and adapt to beat-to-beat intervals.

**Results:**

The algorithms were tested by an independent expert, thus excluding possible author's influence, using all 48 full-length ECG records of the MIT-BIH arrhythmia database. The results were: sensitivity *Se *= 99.69 % and specificity *Sp *= 99.65 % for Algorithm 1 and *Se *= 99.74 % and *Sp *= 99.65 % for Algorithm 2.

**Conclusion:**

The statistical indices are higher than, or comparable to those, cited in the scientific literature.

## Background

The QRS complexes and ventricular beats in an electrocardiogram represent the depolarization phenomenon of the ventricles and yield useful information about their behavior. Beat detection is a procedure preceding any kind of ECG processing and analysis. For morphological analysis this is the reference for detection of other ECG waves and parameter measurements. Rhythm analysis requires classification of QRS and other ventricular beat complexes as normal and abnormal. Real-time ventricular beat detection is essential for monitoring of patients in critical heart condition.

Correct beats recognition is impeded by power-line interference, electromyogram noise and baseline wander often present in the ECG signal.

In long-term monitoring electrode impedance can increase considerably, resulting in very low signal-to-noise ratio, which can make detection practically impossible in a single lead. Therefore, usually two or three leads are used for monitoring [1].

Friesen *et al. *[2] have presented a comparison of nine QRS detection algorithms, based on: i) amplitude and first derivative, ii) first derivative only, iii) first and second derivative, and iv) digital filtering. Daskalov *et al*. [3] applied these algorithms to selected signals containing records with pronounced baseline drift. The results were unsatisfactory, which was probably due to the use of fixed detection thresholds, whereas adaptive ones would be more appropriate.

Poli *et al*. [4] used a generic algorithm for QRS detection. The complexes were emphasized with respect to the rest of the signal by polynomial filters and compared to an adaptive threshold. The authors reported 99.60 % sensitivity (Se) and 99.51 % specificity (Sp) with the MIT-BIH Arrhythmia Database. The method is inapplicable in real-time.

Afonso *et al. *[5] proposed hardware filter banks for ECG signal decomposition, where several parameters were independently computed and combined in a decision rule. The authors reported Se = 99.59 % and Sp = 99.56 % for their real-time, single-channel beat detection algorithm tested with the MIT-BIH Arrhythmia Database.

Dotsinsky and Stoyanov [6] developed a heuristic, pseudo-real-time algorithm for ventricular beat detection for single-channel ECG, based on steep edges and sharp peaks evaluation criteria. They reported Se = 99.04% and Sp = 99.62%, obtained with two channel recordings from AHA and MIT-BIH Arrhythmia Database

Moraes *et al*. [1] combined logically two different algorithms working in parallel – the first has been taken from the work of Englese and Zeelenberg [7] and the other was based on Pan and Tompkins [8], and Ligtenberg and Kunt [9]. Moraes *et al*. [1] reported Se = 99.22 % and Sp = 99.73 % after having excluded records of patients with pacemaker. After excluding a few more recordings 108, 200, 201 and 203, containing high amplitude noise (according to the authors), the statistical indices rises to Se = 99.56 % and Sp = 99.82 %.

Li *et al*. [10] have used wavelet transforms for detection. They reported 0.15 % false detections out of 46 files from the MIT-BIH Arrhythmia Database, but with exclusion of files 214 and 215. In addition, we found some errors in their Table II. After correction, the reported accuracy slightly decreased.

The large variety of QRS detection algorithms, and the continuous efforts for their enhancement, proves that universally acceptable solution has not been found yet. Difficulties arise mainly from the huge diversity of the QRS complex waveforms and the noise and artifacts accompanying the ECG signals.

## ECG databases

All 48 ECG recordings of MIT-BIH Arrhythmia database were used, without exception. Each one has a duration of 30 min and includes two leads – the modified limb lead II and one of the modified leads V1, V2, V4 or V5 [11]. The sampling frequency is 360 Hz with resolution 5 μV/bit. Two cardiologists have annotated all beats. Approximately 70 % of the beats are annotated as *Normal*. Four of the records are from patients with pacemakers.

The American Heart Association (AHA) database was also considered, during the evaluation of the method, mostly due to the fact that it contains patients with premature ventricular beats of contraction of R-over-T type, some of them very difficult to detect because of their closeness to the previous complex. Statistical indices for this database are not derived, because they can be compared with limited number of articles working with AHA. The database consists of 80 recordings: 2-leads, 250 Hz sampling rate and 5 μV/bit resolution.

## Method

The differentiated and summed signals from *L *leads are compared to the absolute value of a threshold *MFR *= *M *+ *F *+ *R *– a combination of three independent adaptive thresholds, where:

• *M *– Steep-slope threshold;

• *F *– Integrating threshold for high-frequency signal components;

• *R *– Beat expectation threshold.

Two algorithms were developed:

*Algorithm 1 *detects at the current beat.

*Algorithm 2 *Pseudo-real-time detection with additional triggering of potentially missed heart beat in the last interval by RR interval analyses.

The algorithms are self-adjusting to the thresholds and weighting constants, regardless of resolution and sampling frequency used. They operate with any number *L *of ECG leads, self-synchronize to QRS or beat slopes and adapt to beat-to-beat intervals.

### Preprocessing

• Moving averaging filter for power-line interference suppression: averages samples in one period of the power-line interference frequency with a first zero at this frequency.

• Moving averaging of samples in 28 ms interval for electromyogram noise suppression – a filter with first zero at about 35 Hz.

• Moving averaging of a complex lead (the sintesis is explained in the next section) in 40 ms intervals – a filter with first zero at about 25 Hz. It is suppressing the noise magnified by the differentiation procedure used in the process of the complex lead sintesis.

### Complex lead

The algorithm operates with a complex lead *Y *of several primary leads *L*. In cases of 12-standard leads, synthesis of the three quasi-orthogonal Frank leads is recommended first [3,12], thus determining the complex lead as a spatial vector. The complex lead is obtained as:



where *Xj(i) *is the amplitude value of the sample *i *in lead *j*, and *Y(i) *is the current complex lead.

The above formula (except the normalizing coefficient 1/L and the absolute value) was initially adopted from the work of Bakardjian [13]. Operating with unsigned (absolute) values proved convenient when dealing with QRSs and extrasystoles having different, for example positive (in one lead) and negative (in the other lead) deflections.

### Adaptive steep-slope threshold – M

• Initially *M *= 0.6*max(Y) is set for the first 5 s of the signal, where at least 2 QRS complexes should occur. A buffer with 5 steep-slope threshold values is preset:

*MM *= [*M*_1_*M*_2_*M*_3_*M*_4_*M*_5_],

where *M*_1 _÷ *M*_5 _are equal to *M*.

• QRS or beat complex is detected if *Yi *≥ *MFR*,

• No detection is allowed 200 ms after the current one. In the interval QRS ÷ QRS_+200*ms *_a new value of *M*_5 _is calculated:

*newM*_5 _= 0.6*max(Yi)

The estimated *newM*_5 _value can become quite high, if steep slope premature ventricular contraction or artifact appeared, and for that reason it is limited to *newM*_5 _= 1.1* *M*_5 _if *newM*_5 _> 1.5* *M*_5_.

The *MM *buffer is refreshed excluding the oldest component, and including *M*_5 _= *newM*_5_. *M *is calculated as an average value of *MM*.

• *M *is decreased in an interval 200 to 1200 ms following the last QRS detection at a low slope, reaching 60 % of its refreshed value at 1200 ms.

• After 1200 ms *M *remains unchanged.

**Figure 1:**
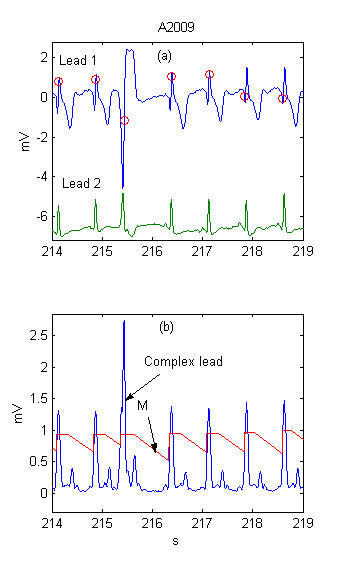
Adaptive steep-slope threshold

The thresholds definitions are presented in more detail with the help of several examples. Two ECG leads are shown in Fig. 1a. Detected QRSs are marked with 'red O' on Lead 1. The summary lead and the steep-slope threshold are represented in Fig. 1b.

### Adaptive integrating threshold – *F*

The integrating threshold *F *is intended to raise the combined threshold if electromyogram noise is accompanying the ECG, thus protecting the algorithm against 'erroneous beat detection'.

Initially *F *is the mean value of the pseudo-spatial velocity *Y *for 350 ms.

With every signal sample, *F *is updated adding the maximum of *Y *in the latest 50 ms of the 350 ms interval and subtracting *maxY *in the earliest 50 ms of the interval.

F = F + (max(Y_in latest 50 ms in the 350 ms interval_) - max(Y_in earliest 50 ms in the 350 ms interval_))/150

The way *F *is updated means that not every sample in the interval is integrated, but just the envelope of the pseudo-spatial velocity *Y*. The weight coefficient 1/150 is empirically derived.

**Figure 2:**
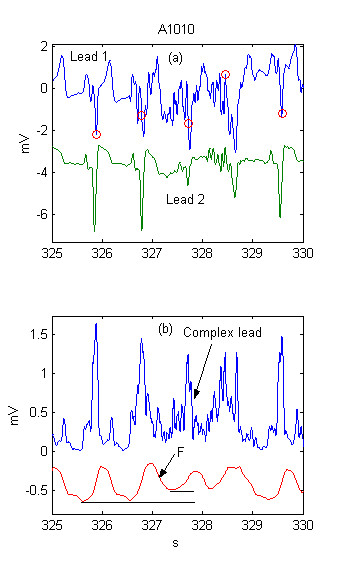
Adaptive integrating threshold

Two ECG leads are shown in Fig. 2a. The pseudo-spatial velocity *Y *and the integrated threshold are presented in Fig. 2b. The correct detection is due to the rise of *F *(hence of *MFR*) with about 0.2 mV. The beat complex is included in the integration process (note the high rise of *F *after any of the complexes), thus making almost impossible a close detection to the previous complex.

### Adaptive beat expectation threshold – *R*

The beat expectation threshold *R *is intended to deal with heartbeats of normal amplitude followed by a beat with very small amplitude (and respectively with very small slew rate). This can be observed for example in cases of electrode artifacts. Conversely to the integrating threshold protecting against erroneous QRS detection, *R *is protecting against 'QRS misdetection'.

A buffer with the 5 last RR intervals is updated at any new QRS detection. *Rm *is the mean value of the buffer.

• *R *= 0 *V *in the interval from the last detected QRS to 2/3 of the expected *Rm*.

• In the interval QRS + Rm * 2/3 to QRS + Rm, *R *decreases 1.4 times slower then the decrease of the previously discussed steep slope threshold (*M *in the 200–1200 ms interval).

• After QRS + Rm the decrease of *R *is stopped.

**Figure 3:**
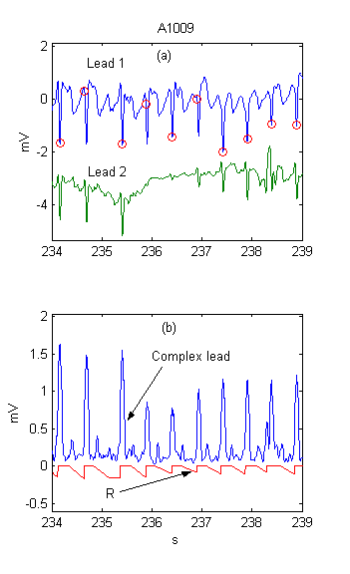
Adaptive beat expectation threshold

The time-course of the beat expectation threshold *R *is shown in Fig. 3. The decrease of *R *(respectively *MFR*) with about 0.2 mV at the fourth QRS allows its detection, despite the lack of complex in Lead 2, which leads to a two-fold decrease of the summary lead amplitude *Y *(Fig. 3b).

### Combined adaptive threshold – *MFR*

**Figure 4:**
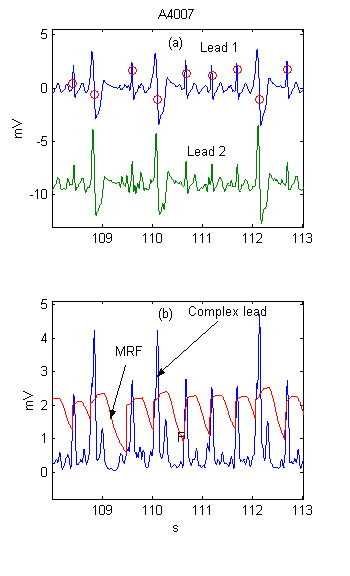
Combined adaptive threshold

The combined adaptive threshold is a sum of the adaptive steep-slope threshold, adaptive integrating threshold and adaptive beat expectation thresholds. (Fig. 4)

*MFR *= *M *+ *F *+ *R*

### Algorithm 2: pseudo-real-time detection with additional triggering of eventually missed heart beat in the last detected RR interval

**Figure 5:**
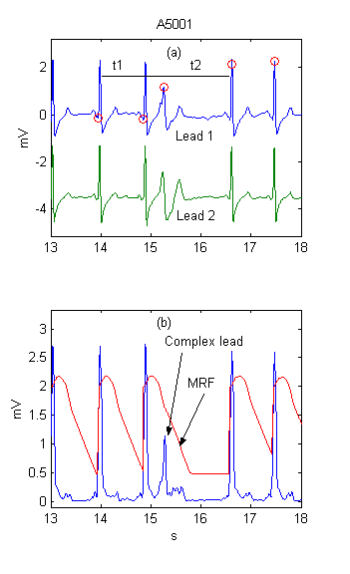
Pseudo-real-time detection with additional triggering of eventually missed heart beat in the last RR interval.

All previous considerations relate to Algorithm 1, which detects a beat at its occurrence. Additional checking for an eventually missed heartbeat is performed by Algorithm 2. Its function is explained by the signal in Fig. 5. The fourth complex at the 15.2 s in Fig. 5b should be missed due to the fact that, *MFR *is greater then the summary lead *Y*.

Let's mark the previous RR interval with *t1 *and the last – with *t2 *(Fig. 5a).

If *t1 *is not shortened, which is tested by logic OR of the 2 conditions t1>Rm OR Rm-t1<0.12*Rm AND in the same time *t2 *is quite long to fulfill the condition abs(t2-2*Rm)<0.5*Rm, the interval is subjected to check for a missed complex.

A test is performed on each of the primary leads where a sharp peak is searched (defined as a product > 4 μV of two signal differences having one central and two lateral points 8 ms apart). If the test is passed, a second one is carried out for the amplitude of the summary lead at that point, which should be bigger then 1/3 of the mean value of the buffer *MM*, in order to define this point as a missed QRS complex.

## Results and discussion

Normally the statistical indices Se and Sp are derived from the following parameters: correctly detected beats TP (true positive), falsely detected beats FP (false positive) and undetected beats FN (false negative). In addition, we used two parameters, adopted from Dotsinsky and Stoyanov [6], as described below.

**Figure 6:**
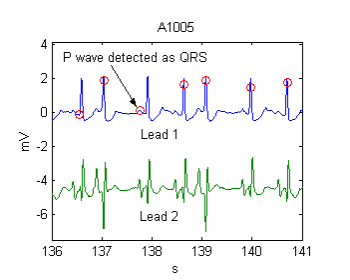
Shifted positive error at the P wave

SP – shifted positive error was introduced in order to explain cases like the one shown in Fig. 6. Here the algorithm made a false positive error before the 3rd QRS and missed the following QRS. Formally, this is a false positive error, immediately followed by a false negative. However, if the time interval between these two detections is reasonably short, for example ≤ 200 ms, we accepted this as one error only, labeled as *Shifted False Positive Error *(SP).

**Figure 7:**
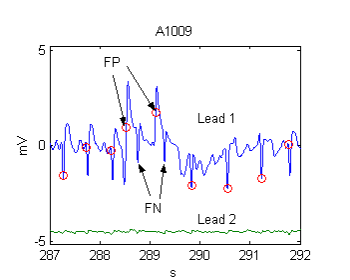
Shifted positive errors, false positive + false negative twins

Another example of SP error as a result of artifacts just before the normal complexes is shown in Fig. 7.

SN – shifted negative error was assumed by the same principle as SP, but in the opposite sense. It also included twin FN+FP error occurring within 200 ms. The first incoming FP or FN error of the shifted is defining it as SP or SN.

The logic of using shifted errors (instead of FP and FN or FN and FP in cases when they appear within 200 ms of each other) is that thus the total number of beats in a record retains its value. Otherwise it would change depending on the type and number of errors and thus impede correct computation of Se and Sp.

The sensitivity Se is calculated by summing FN  SN, while the specificity Sp – by summing FP+SP.



The method was developed in Matlab. All 48 recordings from the MIT-BIH Arrhythmia database, without any exception, were used for testing the two algorithms.

The processed files containing detection marks were automatically compared with the original MIT-BIH annotated beats by specially designed software. It shows all cases where the annotation and detection marks differ by more than 60 ms. These cases were examined by an independent expert, thus excluding possible author's influence.

The results for the two algorithms are presented in Table 1.

**Table 1:** Statistical results for the two algorithms

File	Annotated beats	Algorithm 1					Algorithm 2				
			
		TP	FN	FP	SN	SP	TP	FN	FP	SN	SP
100	2273	2273	0	0	0	0	2273	0	0	0	0
101	1863	1862	1	4	0	0	1862	1	4	0	0
102	2187	2187	0	0	0	0	2187	0	0	0	0
103	2084	2062	2	54	11	9	2065	0	58	12	7
104	2212	2211	1	0	0	0	2211	1	0	0	0
105	2567	2543	2	35	8	14	2544	2	36	8	13
106	2027	2017	1	1	0	9	2018	0	1	0	9
107	2137	2135	2	0	0	0	2137	0	0	0	0
108	1763	1664	2	40	3	94	1674	1	42	3	85
109	2532	2521	11	1	0	0	2527	5	0	0	0

111	2124	2124	0	0	0	0	2124	0	0	0	0
112	2539	2539	0	0	0	0	2539	0	0	0	0
113	1797	1797	0	0	0	0	1797	0	0	0	0
114	1879	1879	0	0	0	0	1879	0	0	0	0
115	1953	1951	0	4	1	1	1952	0	4	0	1
116	2412	2389	22	2	0	1	2392	19	2	0	1
117	1535	1535	0	0	0	0	1535	0	0	0	0
118	2275	2275	0	0	0	0	2275	0	0	0	0
119	1987	1987	0	0	0	0	1987	0	0	0	0
121	1863	1863	0	0	0	0	1863	0	0	0	0

122	2476	2476	0	0	0	0	2476	0	0	0	0
123	1518	1516	2	0	0	0	1516	2	0	0	0
124	1619	1617	2	0	0	0	1619	0	0	0	0
200	2601	2549	9	39	18	25	2552	6	41	20	23
201	1963	1902	60	0	0	1	1902	60	0	0	1
202	2136	2130	6	0	0	0	2130	6	0	0	0
203	2978	2901	71	13	3	3	2911	62	27	3	2
205	2656	2652	4	0	0	0	2652	4	0	0	0
207	1862	1860	2	0	0	0	1862	0	1	0	0
208	2954	2937	14	7	2	1	2939	11	7	2	2

209	3004	3004	0	1	0	0	3004	0	1	0	0
210	2647	2591	56	1	0	0	2603	44	1	0	0
212	2748	2748	0	0	0	0	2748	0	0	0	0
213	3551	3548	3	0	0	0	3550	1	0	0	0
214	2260	2258	1	1	1	0	2256	4	1	0	0
215	3362	3362	0	0	0	0	3362	0	0	0	0
217	2208	2204	3	0	0	1	2205	2	0	0	1
219	2154	2153	1	0	0	0	2153	1	0	0	0
220	2048	2048	0	0	0	0	2048	0	0	0	0
221	2427	2426	1	0	0	0	2426	1	0	0	0

222	2483	2480	2	0	0	1	2482	0	0	0	1
223	2595	2585	10	0	0	0	2590	5	0	0	0
228	2053	2053	0	0	0	0	2053	0	1	0	0
230	2256	2256	0	0	0	0	2256	0	0	0	0
231	1886	1886	0	0	0	0	1886	0	0	0	0
232	1767	1766	0	12	0	1	1766	0	12	0	1
233	3076	3073	3	0	0	0	3074	2	0	0	0
234	2753	2753	0	0	0	0	2753	0	0	0	0

Sum	110050	109548	294	215	47	161	109615	240	239	48	147

Of all 110050 annotated beats ('unknown' or 'questionable' were excluded from the study), true detected are 109548 for Algorithm 1 and 109616 for Algorithm 2. The statistical indices are:

**Algorithm 1**: Se = 99.69 %, Sp = 99.66 %;

**Algorithm 2**: Se = 99.74 %, Sp = 99.65 %.

The standard way of Se and Sp calculation,



considering the joint SP and SN errors as two separate errors gives the following results:

**Algorithm 1**: Se = 99.54 %, Sp = 99.61 %;

**Algorithm 2**: Se = 99.60 %, Sp = 99.60 %.

Algorithm 2 improved the sensitivity by 0.05 % (0.06 % for the standard evaluation) as a result of decreased number of undetected beats. This result can be observed for example in recordings 109,203, 210 and 223, where the additionally detected beats are respectively 6, 9, 12 and 5. The performance of both algorithms was especially tested with the file A5001 from the AHA containing R-over-T premature ventricular complexes, very close to the previous normal QRS complex (Fig. 5a). An improvement of 74 undetected by Algorithm 1 R-on-T complexes was observed. The detection of such premature ventricular complexes occurring at the time of ventricular repolarization was considered important, having in mind possible risk of ventricular fibrillation triggering by R-on-T events.

## Conclusions

The proposed algorithms for real-time and pseudo-real-time implementation are adaptive, independent of thresholds and constants values. They are self-synchronized to the QRS steep slope and the heart rhythm, regardless of the resolution and sampling frequency used. Due to the integration threshold, the algorithms are practically insensitive to electromyogram and similar high-frequency noise.

The algorithms can operate with one, two or more leads, using a combined lead signal derived from the sum of absolute values of the differentiated lead signals.

The statistical indices are higher than, or comparable to those, cited in the scientific literature.
